# Comparative structural analyses and nucleotide-binding characterization of the four KH domains of FUBP1

**DOI:** 10.1038/s41598-020-69832-z

**Published:** 2020-08-10

**Authors:** Xiaomin Ni, Stefan Knapp, Apirat Chaikuad

**Affiliations:** 1 Institute of Pharmaceutical Chemistry, Goethe-University Frankfurt, 60438 Frankfurt, Germany; 2 Structural Genomics Consortium, BMLS, Goethe-University Frankfurt, 60438 Frankfurt, Germany; 3 German Translational Cancer Network (DKTK), Frankfurt/Mainz Site, 60438 Frankfurt, Germany

**Keywords:** Biochemistry, Structural biology

## Abstract

The FUBP1-FUSE complex is an essential component of a transcription molecular machinery that is necessary for tight regulation of expression of many key genes including c-Myc and p21. FUBP1 utilizes its four articulated KH modules, which function cooperatively, for FUSE nucleotide binding. To understand molecular mechanisms fundamental to the intermolecular interaction, we present a set of crystal structures, as well ssDNA-binding characterization of FUBP1 KH domains. All KH1-4 motifs were highly topologically conserved, and were able to interact with FUSE individually and independently. Nevertheless, differences in nucleotide binding properties among the four KH domains were evident, including higher nucleotide-binding potency for KH3 as well as diverse nucleotide sequence preferences. Variations in amino acid compositions at one side of the binding cleft responsible for nucleobase resulted in diverse shapes and electrostatic charge interaction, which might feasibly be a contributing factor for different nucleotide-binding propensities among KH1-4. Nonetheless, conservation of structure and nucleotide-binding property in all four KH motifs is essential for the cooperativity of multi KH modules present in FUBP1 towards nanomolar affinity for FUSE interaction. Comprehensive structural comparison and ssDNA binding characteristics of all four KH domains presented here provide molecular insights at a fundamental level that might be beneficial for elucidating the mechanisms of the FUBP1-FUSE interaction.

## Introduction

Far upstream element (FUSE) binding protein 1 (FUBP1 or FBP1) is a multifunctional single-stranded DNA- (ssDNA) and RNA-binding protein, which acts as a master regulator of diverse cellular processes including transcription, mRNA stability and translation as well as RNA splicing^1–3^. It is best known for its role as a positive regulator of c-Myc oncoprotein mediated by the interaction with the supercoiled, A/T-rich non-coding strand of FUSE located upstream of the *c-Myc* promoter^4–6^ which leads to the recruitment and activation of transcription factor TFIIH that enhances transcription of *c-Myc* gene^7^. FUBP1 also recruits the FBP interacting repressor (FIR) to form an inhibitory complex with FUSE and TFIIH, which suppresses *c-Myc* transcription. The interplay between FUSE, FUBP1 and FIR in this molecular machinery is essential for controlling the level and timing of c-Myc expression^5,8^. Apart from this, other roles of FUB1 in several post-transcriptional events have also been demonstrated. For instance, FUBP1 is often found in association with spliceosomal complexes, and depending on context can either promote or suppress RNA splicing^3,9–11^. Mediated by modulating expression of specific target genes including c-Myc^4^, p21^12^ and Usp29^13^, FUBP1 has been identified as a potent pro-proliferative and anti-apoptotic factor that is essential for many biological processes in diverse cell types, such as hematopoietic stem cell maintenance and survival^14,15^ as well as neuronal differentiation and tumour suppression in the nervous system^9^.

Due to the pivotal roles in regulating multiple cellular processes, an alteration of FUBP1 has a strong link to the development of a number of diseases. FUBP1 has been described paradoxically either as an oncoprotein or a tumour suppressor. Loss of function due to mutations in *fubp1* gene is often detected in patients with central nervous system diseases and intestinal cancer^1,16,17^. On a contrary, overexpression of FUBP1 is also often found and appears as an emerging suspect in hematologic disorders and a growing number of cancers and solid tumours, including breast and colon cancers as well as nasopharyngeal and clear cell renal cell carcinoma^18–20^. High level of FUBP1 typically leads to an upregulation of c-Myc oncogene and deregulation of the fine-tuned expression of targeted proteins, forming one of main molecular mechanisms of pathogenesis in most cases. Nevertheless, other molecular consequences independent of c-Myc have been proposed as a contributing factor for the development of some diseases. This includes for example facilitating the replication of hepatitis C virus^21^ as well as suppressing the expression of cell cycle inhibitors like p21 through mRNA binding^12^, both of which are associated with the formation and development of hepatocellular carcinoma.

Central to its single-stranded nucleic acid (ssNA) interacting function of human FUBP1 is a tandem of four K homology (KH) motifs, namely KH1, KH2, KH3 and KH4, which is embedded in the middle region in between the self-regulatory and transactivation domains at the N- and C- termini, respectively^22,23^. Typically, eukaryotic KH domains are small (~ 70 amino acids) and share a highly conserved topology, which is distinct from that of the prokaryotic proteins^22,24^. A common property in recognition of up to four nucleotides is facilitated by a ‘minimal KH motif’ that constructs an ssNA hydrophobic binding cleft formed on one side by two short consecutive helices (α1 and α2) and the GXXG-motif-containing connecting loop and on the other side by the domain’s β-sheet and the variable loop that links the β2 and β3. Nevertheless, different KH domains have been shown to exert different ssNA specificities and some can further form non-specific contacts with additional flanking nucleotides^22,25^. For instance, the third KH domain of KSRP (known also as FUBP2) prefers a G-rich sequence, while hnRNP K KH3 and PCBP KH motifs prefer a C-rich signature^25–29^. Binding affinities between individual KH domain and ssNA are however moderate, typically in a low to several hundred micro molar K_D_ range^26,27,30^, and therefore cooperativity of multiple KH modules in a protein potentially forms an essential mechanism for achieving tight interactions^6,27,30^.

For FUBP1, the early NMR solution structure of the paired KH3 + KH4 domain in complex with ssDNA has unveiled not only their evolutionarily highly conserved KH topology, but provided first molecular details for nucleotide recognition^31^. Despite using commonly the GXXG motif for the interaction with the ssDNA backbone, slight changes of amino acid compositions within the β sheet and the variable loop could contribute to different preferences of KH3 and KH4 for 5′d-TTTT and 5′d-ATTC, respectively. Later, ssDNA-binding studies using the paired KH domains and the systematic evolution of ligand by exponential enrichment (SELEX) technique have identified a number of potential binding sites on c-Myc FUSE, all of which share commonly a TGT footprint core^6^. However, KH1-4 might exhibit different preferences on nucleotide sequences; T(T/C)GT for KH2, KH3 and KH4 and (T/G)TG(T/C) for KH1. Nonetheless, these early studies have suggested consistently that all four KH domains of FUBP1 likely function as articulated modules and exploit hierarchical mechanisms to specify their binding sites on FUSE^6,31^.

The structural and mechanistic knowledge from the previous studies have contributed to our understanding on the ssDNA-binding function of the FUBP1 KH motifs. However, it remains unclear whether each KH domain might possess intrinsic properties that might influence binding affinities and nucleotide specificities. Here, we presented a set of crystal structures and ssDNA-binding characterization of KH1, 2, 3 and 4, revealing a highly conserved KH fold that is essential for their individual abilities to interact with a short FUSE ssDNA fragments. Comparative structural analyses and ssDNA binding studies demonstrated high variation of amino acids compositions essentially within the GXXG motif, α1, the central β sheet and β2–β3 variable loop led to different characteristics of their nucleotide binding pockets. These alterations might feasibly be accounted for the different ssDNA-binding properties among KH1-4, demonstrated by a higher binding affinity for KH3 and diverse nucleotide sequence preferences. Nevertheless, these four KH motifs of FUBP1 with conserved structure and nucleotide-binding property likely function cooperatively enabling a nanomolar affinity for FUSE interaction. The knowledge from our comprehensive studies would enable further understanding of the molecular mechanisms of the FUSE-interacting function of FUBP1.

## Results

### FUBP1 KH1-4 adopted commonly a highly conserved KH topology

**Figure 1:**
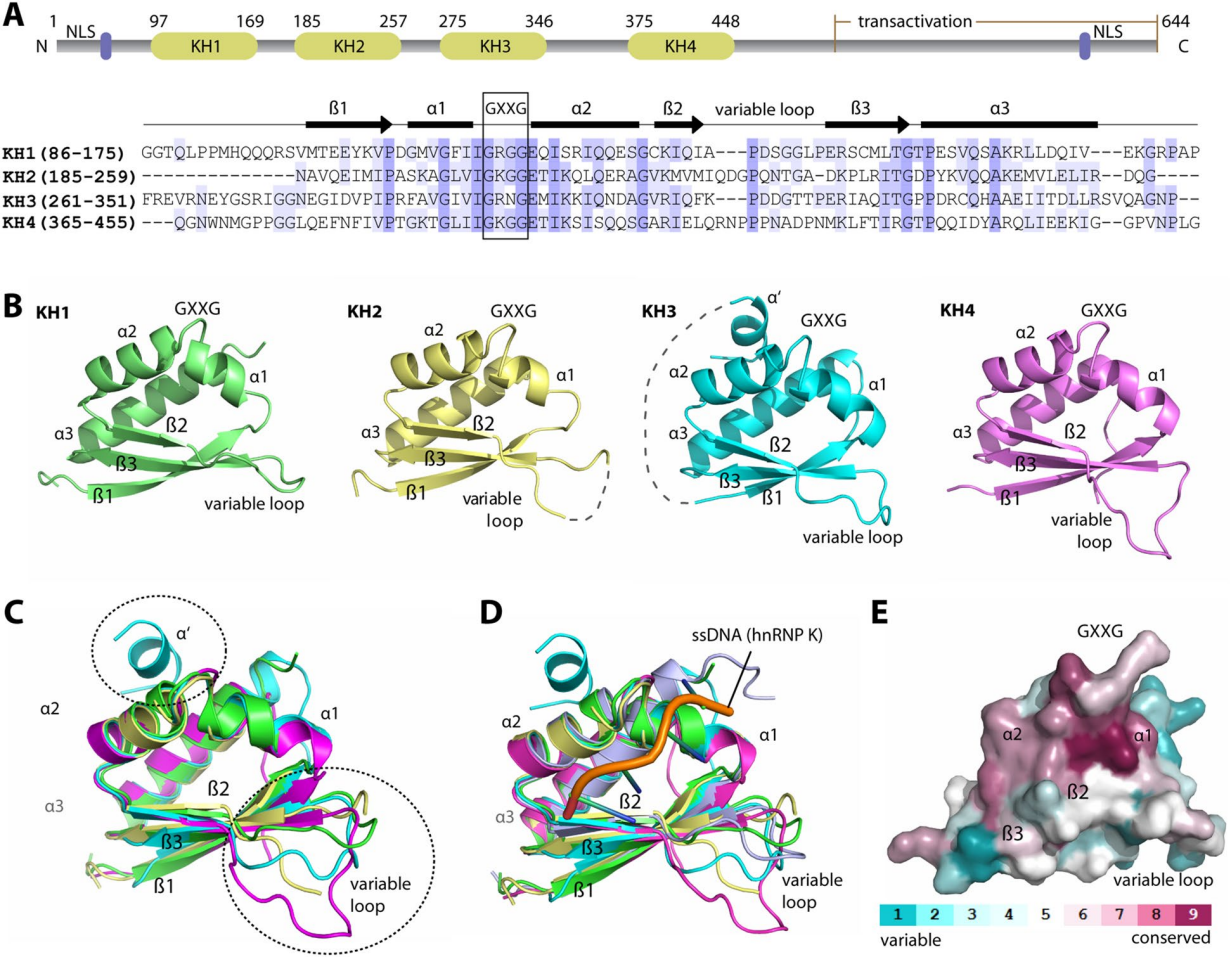
Crystal structures of FUBP1 KH1, 2, 3 and 4. (**A**) Domain architecture of FUBP1 (top) and structure-based sequence alignment of the KH1-4 constructs used for crystallisation (bottom). The conserved amino acids are highlighted in blue with the GXXG motif in the black, square box. (**B**) Overall structures of KH1 (green), KH2 (yellow), KH3 (cyan) and KH4 (magenta). (**C**) Structural superimposition of FUBP1 KH1-4 revealed conserved overall topology, except for the conformation of the β2-β3 variable loop and the α′ extra helix in KH3 (dashed circles). (**D**) Superimposition of FUBP1 KH1-4 and the ssDNA-bound hnRNP K KH3 (light blue with the bound ssDNA shown in orange ribbon, PDB code: 1zzi). (**E**) Mapping of amino acid conservation calculated by ConSurf^44^. The surface representation is shown with a similar orientation to that in (**C**). Figures were created using PyMOL software (https://pymol.org/).

In the search for optimal constructs for crystallisation, we first performed sequence alignment to identify an optimal domain boundary for each KH motif of FUBP1. All four KH domains shared sequence identities of 33–38% for the central β-sheet and helical core. However, this was in contrast to high sequence differences observed for the linker regions between the domains (Supplementary Figure S1). Based on this, we generated a series of truncated constructs by varying the length of the linker region that flanks the N- and C-termini of the central core (Supplementary Figure S1B), from which constructs with good expression levels led to suitable protein crystals and subsequently to successfully determined crystal structures of KH2, KH3 and KH4 at 1.9—2.0 Å resolution (Fig. 1A,B).

Together with the available KH1 crystal structure (PDB ID: 4lij) the three structures determined here provide a complete structural characterization of all four KH domains of FUBP1. Comparison of the four structures showed that they all adopted an evolutionarily conserved eukaryotic KH-domain architecture, consisting of a three-stranded β-sheet packed on one side by three helices (Fig. 1B,C). Such topology highly resembled that of KH domains from other proteins, such as hnRNP K^25^ and KSRP^26^, as demonstrated by low superimposition rmsd (root mean squares deviations) values of 1.1–2.4 Å (Fig. 1D). Comparison among four FUBP1 KH domains revealed that the highly conserved residues from α1-α2 region harbouring the canonical GXXG motif within the connecting loop clustered within one side of their DNA binding pockets (Fig. 1E). Nevertheless, some differences were noted for the variable loop between the β2 and β3, which was located at the other side of the DNA binding groove (Fig. 1E). This loop differed not only in their lengths, but as a consequence in their conformations (Fig. 1C). In addition, we observed also a unique, additional small α′ helix at the N-terminus of KH3 (Fig. 1B). However, this structural element might be induced by the crystal packing as it existed only in one of the two molecules in the asymmetric unit.

### KH1-4 can interact individually with ssDNA

**Figure 2:**
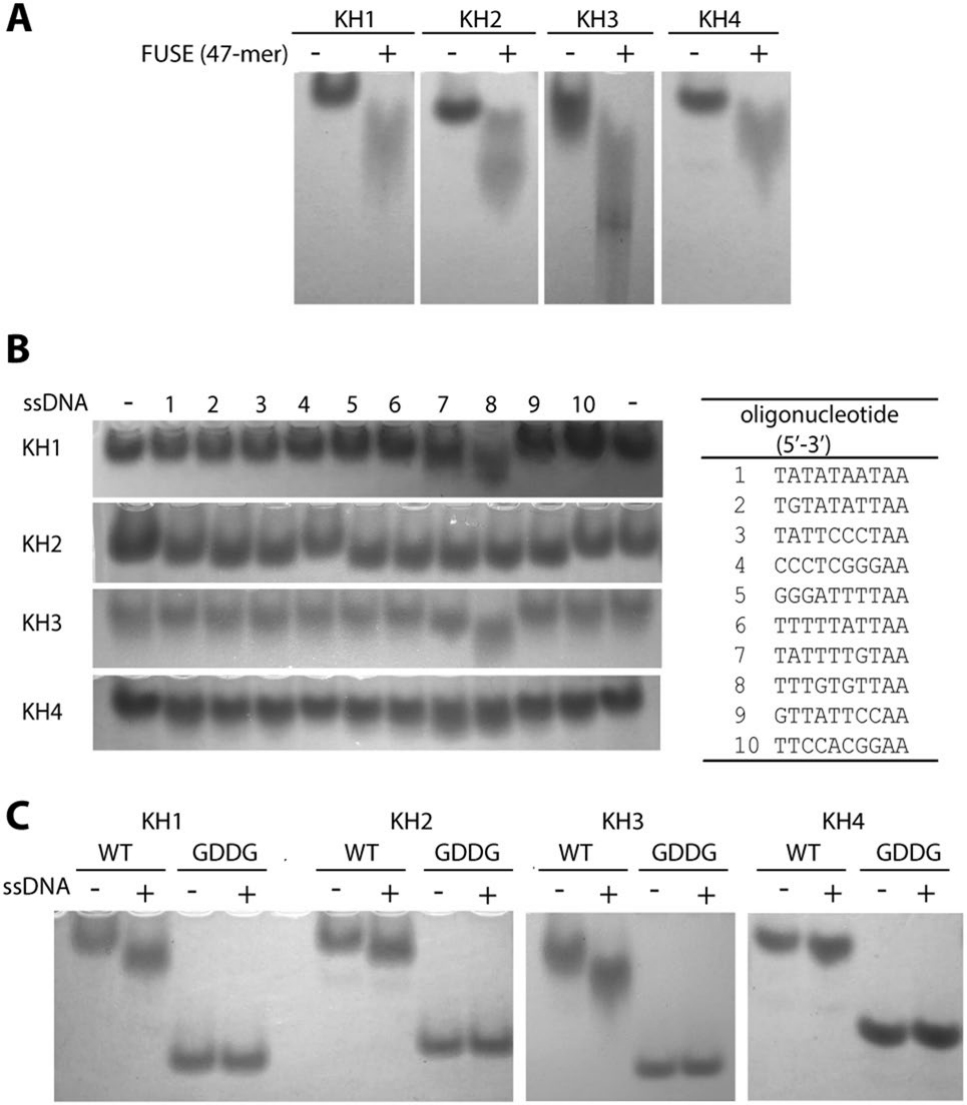
Native gel mobility shift analyses for the interactions between single KH1-4 and c-Myc FUSE ssDNA. (**A**) All single KH1-4 demonstrates migration shifts upon incubating with 47mer FUSE. (**B**) The migration shift patterns of single KH1-4 with various FUSE fragments (right table) reveal potential binding preference of KH1 and 3 with ssDNA fragment 8, and potentially binding of KH2 and 4 with nearly all ssDNA, except fragment 4 and 10. (**C**) Migration shift analyses of the wild-type KH1-4 and their corresponding GDDG mutants with the selected ssDNA (fragment 8 for KH1 and 3, and fragment 7 for KH2 and 4).

Due to their structural conservation as well as the highly conserved and feasibly intact DNA binding pockets, we next questioned whether each KH domain could potentially bind to ssDNA independently. First, we investigated their interactions with the 47-mer c-Myc FUSE (5′-ATGTATATTCCCTCGGGATTTTTTATTTTGTGTTATTCCACGGCATG-3′), which is known to bind to full-length FUBP1^1,6,31^. Native gel mobility shift assays indeed showed a significant shifts of all single KH1-4 protein bands upon incubating with this ssDNA (Fig. 2A), indicating an ability of all four KH protein modules to interact with c-Myc FUSE individually.

We next aimed to investigate whether each KH domain might prefer specific interacting sites, and therefore tested the ability of the 4 individual KH domains to bind to a series of randomly-truncated 10-mer c-Myc FUSE fragments that harboured an AA-double nucleotide on their 3′ ends in order to increase the migration of the proteins in native gels. Interestingly, we observed different migration profiles among KH1-4 (Fig. 2B). Although not significantly pronounced, both KH1 and KH3 showed a clear shift with the ssDNA fragment 8 (TTTGTGTTAA), which contained a ‘TGT’ core predicted previously as an optimal binding site for FUBP1 KH domains^6^. Nevertheless, potential shifts of KH1 and KH3 were also evident for other fragments such as number 7, but to a much lesser extent. In contrast, the migration patterns of KH2 and KH4 were rather ambiguous with some slight shifts observed for nearly all nucleotides, except fragment 4 and 10 that had a high GC-rich content. Interestingly, the potential interactions of KH2 and 4 with ssDNA 5–8, which shared the presence of a triple-repeated deoxythymidine (TTT), was consistent with the previous suggestion on the preference of FUBP1 for T-enriched sequence^6^.

To further verify and confirm the DNA binding preferences of each KH domain, we mutated the GXXG motif of the KH domains to GDDG, which is known to disrupt their interactions with ssDNA through modulating charges on the binding surface^32^. As expected, in contrast to the wild type, all mutants did not show a shift in migration on native gels upon incubation with the selected ssDNA (Fig. 2C). These overall results suggested therefore that all four KH motifs of FUBP1 interacted with c-Myc FUSE ssDNA independently and individually, and KH1 and 3 might exert potential preference for ‘TTTGTGTT’ sequence while KH2 and KH4 might have broader binding specificity.

### Individual KH domains exhibited only weak binding affinities to ssDNA.

**Figure 3:**
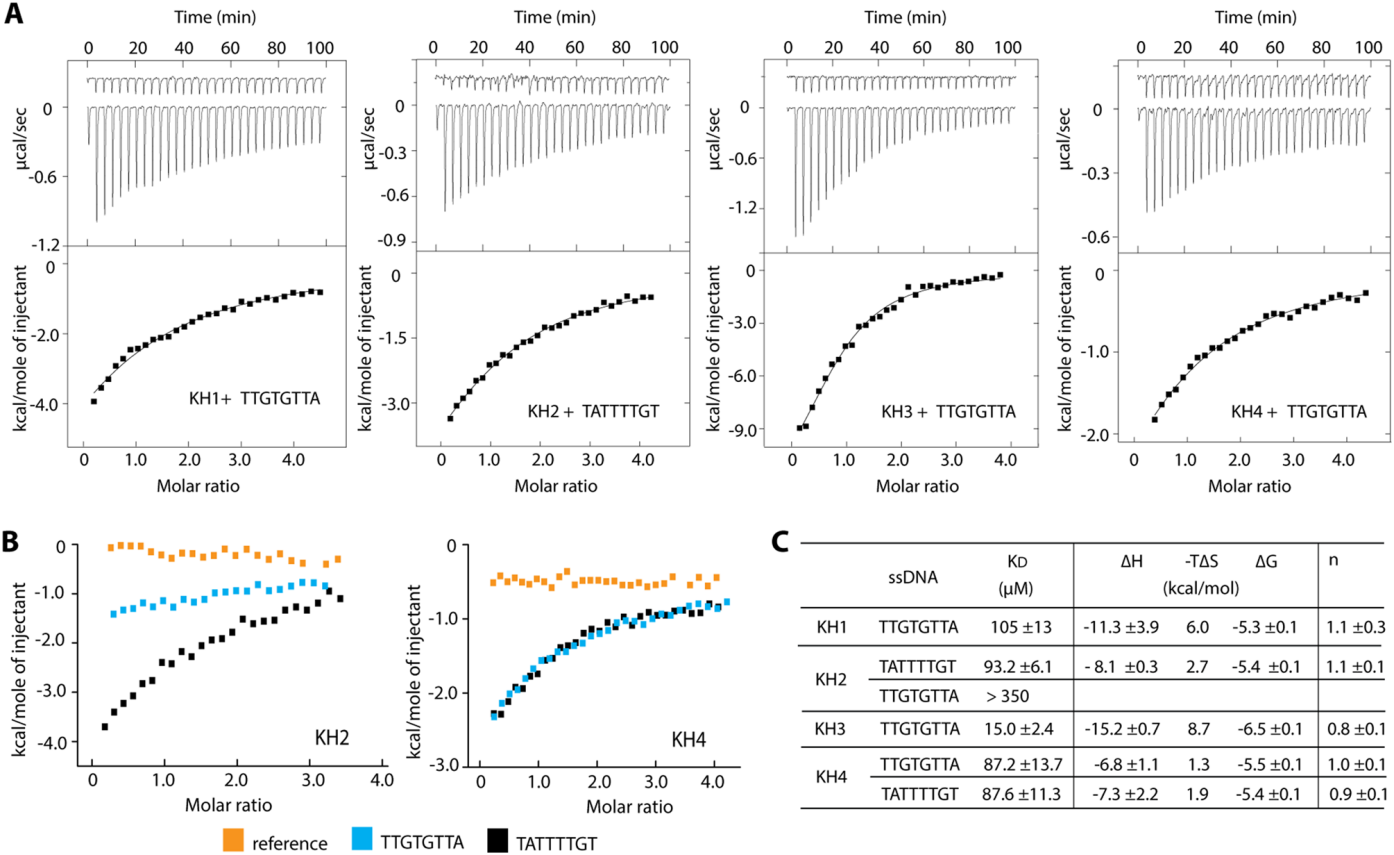
Binding affinities of each KH domain with ssDNA determined by ITC. (**A**) The ITC titration for the interactions between single KH domain and the selected ssDNA. The isotherms of the raw titration heat of the references (protein into buffer, top) and the protein-ssDNA binding (bottom) are shown in the upper panels, while the lower panels are the normalized binding heat with the single-site fitting calculated after the subtraction with the protein dilution heat. (**B**) The ITC normalized binding heat of the interactions between KH2 or KH4 with two ssDNA fragment. The reference refers the dilution heat of the proteins. (**C**) Summary of the average binding constants (K_D_) and thermodynamic parameters calculated from two repeats.

We next performed isothermal titration calorimetry (ITC) to quantify the affinities of the interactions between KH1-4 and their preferred ssDNA. Based on our native gel mobility shift analysis and the previously suggested ideal binding sites containing either the TGT footprint or a T-rich sequence, we chose to perform ITC with two ssDNA fragments; i) TTGTGTTA (FUSE fragment 8) for all four KH domains and ii) TATTTTGT (FUSE fragment 7) for KH2 and KH4. Overall, the ITC results demonstrated that all four single KH domains interacted to the ssDNA with moderate affinities in a low micromolar range (Fig. 3A–C). By comparison, KH3 interestingly exhibited the strongest binding with the K_D_ of 17 µM, which was at least fivefold stronger than that observed for KH1, 2 and 4 (Fig. 3C). In addition, we observed interestingly also that KH2 preferred the T-rich fragment 7 over the TGTG-core fragment 8, which was in contrast to KH4 that showed no preference for both ssDNA (Fig. 3B,C). These cumulative results suggested therefore that each KH domains of FUBP1 exerted different nucleotide binding properties. Nevertheless, although highly estimated due to the nature of weak binding, all KH1-4 shared a common thermodynamic signature of favourable enthalpy and unfavourable entropy for their interactions with ssDNA.

### Similarities and dissimilarities of the ssDNA binding pockets of KH1-4

**Figure 4:**
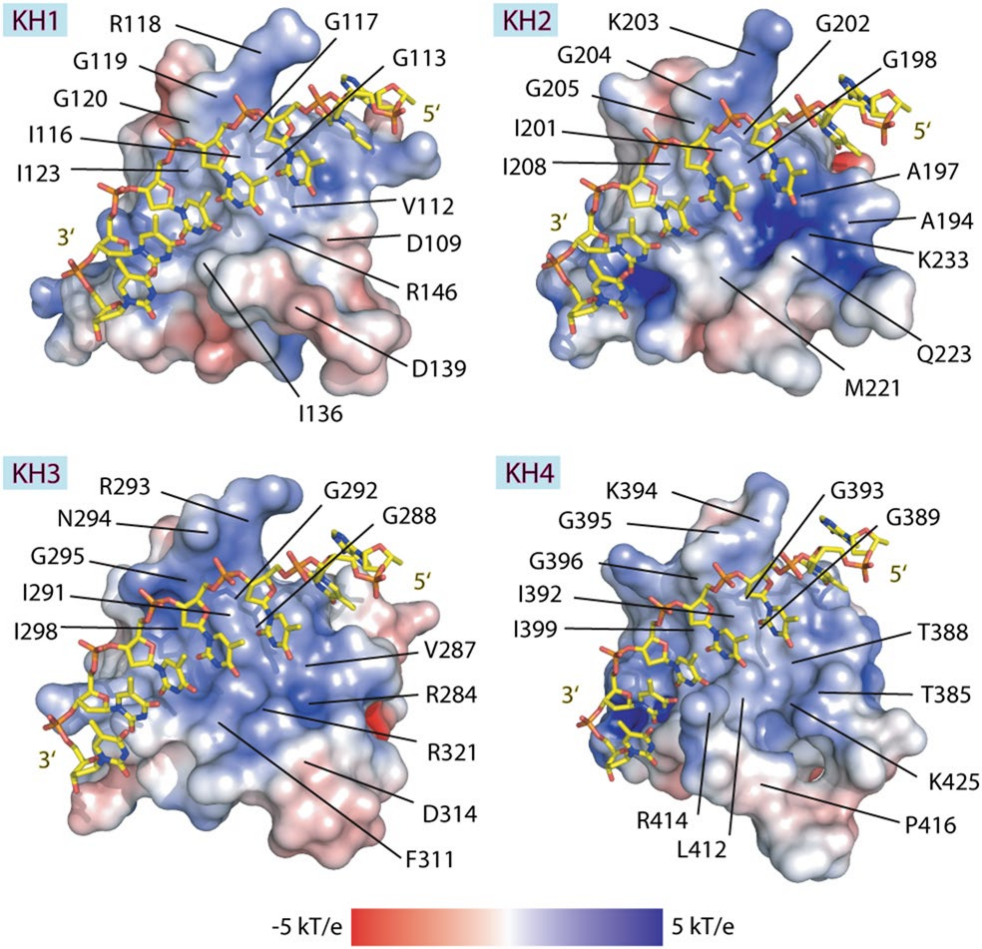
Comparison of the nucleotide binding pockets of FUBP1 KH1-4. Shown are electrostatic potentials of the nucleotide binding surface and ssDNA (ATTTTTT, stick representation) modelled based on the previous NMR structure of ssDNA-complexed KH3 + KH4 (pdb id: 1j4w). Figures were created using PyMOL software (https://pymol.org/).

Despite the uniform propensity of all four KHs for ssDNA interactions, potential differences in nucleotide preferences and a unique increase in the ssDNA binding affinity of KH3 prompted us to investigate the molecular details of their binding pockets and potential molecular basis responsible for such distinct characteristics. Since our attempts on the ssDNA-KH complex crystallisation was unsuccessful, we therefore modelled a single-stranded nucleotide into the apo-structures using the previous NMR model of the ssDNA-complexed with KH3 + KH4^31^. Comparative analyses of the binding site features revealed both similarities and dissimilarities (Fig. 4). First, the binding pockets of all FUBP1 KH domains possessed commonly an overall positively-charged property, likely compatible with recruiting negatively-charged DNA. However, electrostatic potentials of the binding surface were observed to be different. Another common feature was high conservation of amino acid compositions at the upper part of the pocket responsible for interacting with the ssDNA phosphate and deoxyribose backbone. This included the GXXG motif as well as its adjacent cluster of hydrophobic residues such as isoleucine and glycine (e.g. G288, I291 and I298 in KH3). This amino acid signature is conserved also in other KH domains, suggesting its importance in the recognition of ssDNA sequences (Supplementary Figure S2).

In contrast to the upper part of the binding pocket, considerable variations of the amino acid compositions within α1 and the β2-β3 connecting loop led to the remarkably distinct properties of the nucleotide binding pocket at the lower half, which is responsible for interacting with ssDNA nucleobases. For instance, a longer loop together with the presence of small hydrophobic (A194 and A197) or polar residues (T385 and T388) in α1 of KH2 and 4, respectively, resulted in relatively open binding surface with strong positively-charged electrostatic potentials. This was in contrast to the slightly more compact with less positively-charged pocket of KH1, constructed by its shorter loop and acidic aspartate residues (D139 and the α1 D109). Of particular note was KH3, of which the short and compact β2-β3 variable loop and the presence of α1 R284 resulted in the more-enclosed, positively-charged binding groove, which was likely enabled also by the unique glycine-to-asparagine substitution in the GRNG motif at the upper part of the pocket.

### The unique GRNG motif did not contribute to an increase in ssDNA binding affinity in KH3

**Figure 5:**
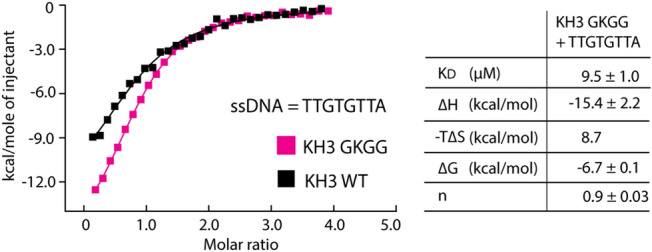
Comparison of the ssDNA binding in wild type KH3 and the GKGG mutant. Superimposition of the ITC integrated heat of the interaction between the ssDNA fragment and wild type KH3 (black) and the GKGG mutant (purple) is shown on the left panel, and summary of the average binding constants (K_D_) and thermodynamic parameters for the mutant calculated from two repeats is shown in the right table.

Unlike the GXGG motif in KH1, 2 and 4, the presence of asparagine in the GRNG motif of KH3 is rather unique. Since this motif was essential for ssDNA binding^31,32^, we questioned whether this substitution might be a contributing factor to the distinctly stronger ssDNA-binding affinity observed for KH3. We therefore generated the KH3 mutant harbouring the GKGG motif resembling that of KH2 and KH4, and measured ssDNA binding using ITC. The results showed that the mutation did not exhibit an adverse effect, yet on a contrary slightly enhanced binding to ssDNA of ~ 1.7 fold was observed (Fig. 5). This suggested therefore that the unique glycine-to-asparagine substitution was unlikely responsible for an increase in the nucleotide-binding affinity in KH3.

### Multiple KH motifs cumulatively contribute towards high affinity binding of FUBP1 to FUSE

**Figure 6:**
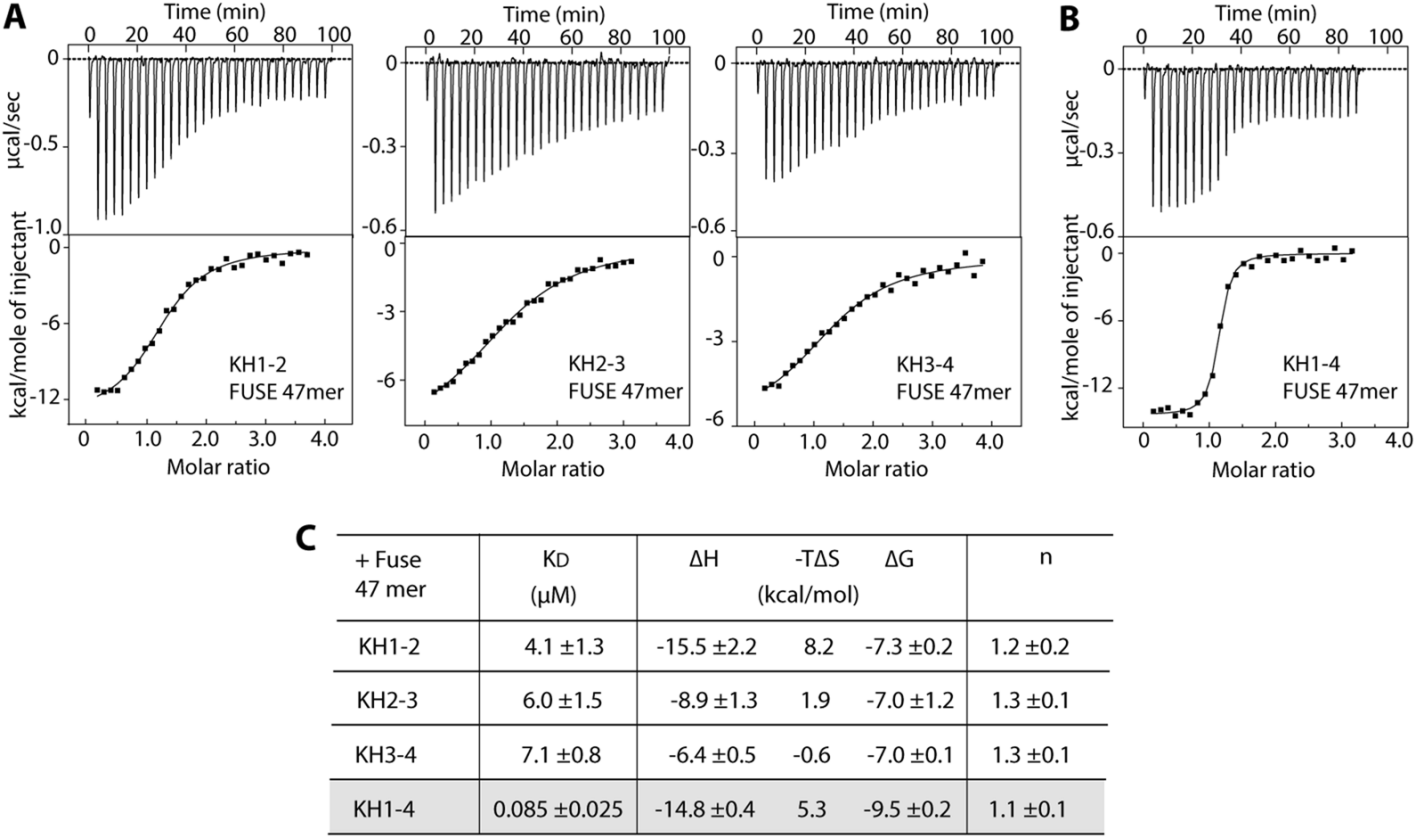
Binding of multi-KH domains and KH1-4 full-length protein to 47-mer FUSE. (**A**) ITC titrations for the interactions between three tandem paired KH domains, including KH1-2, KH2-3 and KH3-4, and 47-mer FUSE ssDNA (5′-ATGTATATTCCCTCGGGATTTTTTATTTTGTGTTATTCCACGGCATG-3′). (**B**) The ITC titration for the interaction between KH1-4 FUBP1 and the 47-mer FUSE DNA. (**C**) Summary of the binding constants (K_D_) and thermodynamic parameters calculated from the average values of two repeats.

We next investigate the importance of conservation of structure and nucleotide-binding property in all four KH motifs towards FUBP1-FUSE interaction. First, we assessed the binding affinities of three tandem paired KH proteins, including KH1-2, KH2-3 and KH3-4, to 47-mer FUSE. The ITC results demonstrated consistently low micromolar binding affinities for all cases (K_D_ values of ~ 4–7 μM) (Fig. 6A), which were ~ 2–15-fold increase compared to the affinities determined for single KH domain. This suggested that increasing the number of KH domain led to a dramatic increase in binding affinities. We thus characterized the interaction between full KH1-4 FUBP1 to FUSE, and indeed observed a remarkable tight binding with a K_D_ of ~ 85 nM (Fig. 6B,C) consistent with other reports^8,33^. This results indicates that all KH motifs of FUBP1, each of which can bind nucleotide independently, likely contribute towards high affinity for FUSE binding.

## Discussion

The pivotal role of FUBP1 as a master regulator that controls transcriptions of key proteins such as c-Myc and p21 is mediated by the FUSE-interacting function of its four KH domains. Previous studies on the ssDNA binding activities of the KH motifs have predicted the sequence signatures of ideal binding sites as well as feasible cooperativity and hierarchical binding mechanisms^6,31^. Here, we presented a comprehensive structural analysis and ssDNA-binding characterization of each KH domain, which provided further molecular insights into the mechanistic details at the fundamental level of FUBP1-FUSE interaction.

KH domain are evolutionary highly conserved and typically share significant sequence similarity in the motif regions. Prokaryotic and eukaryotic KHs have distinct topologies, which nevertheless are highly conserved within their subclasses, essential for a common nucleotide binding function^22,24^. Consistently, our sequence analyses and crystal structures demonstrated that all four KH domains of FUBP1 shared the evolutionarily conserved eukaryotic KH characteristics. The observed abilities of all single KH1-4 to interact with ssDNA individually was in agreement with previous reports demonstrating the capability of a single KH motif for nucleotide binding^25,29,34^. In addition, we demonstrated that multiple KH motifs enabled a dramatic increase in FUSE binding affinities, exemplified by the K_D_ of 85 nM for the full KH1-4 tandem. Overall, these results suggested therefore that all four KH motifs of FUBP1 contain a highly conserved and complete protein module that can bind to nucleotide independently. Such conservation of structure and property is likely important for cooperativity of all four KH domains towards high affinity interaction between FUBP1 and FUSE.

Consistent with the previous observation^6^, ssDNA-binding characterization using FUSE fragments predicted that all four FUBP1 KH domains feasibly prefer the sequence signatures of either TGT or T-rich footprints. Despite exhibiting similarly moderate affinities in low micromolar range, discrepancies in the ssDNA-binding properties of KH1-4 were evident. This included, for example, more than fivefold increase in an affinity for the ssDNA interaction in KH3, as well a greater degree of ssDNA specificity of KH1 and KH3 than KH2 and KH4, the latter of which showed the lowest degree of nucleotide preferences. These different characteristics correlates with previous suggestions of potential diverse roles of each KH motif towards the central nucleotide binding function of FUBP1^31,35^.

Our analyses of potential molecular basis for the dissimilar nucleotide binding properties revealed that a subtle change in amino acid composition within the GXXG signature, exemplified by the unique glycine-to-asparagine substitution in KH3, might not be sufficient as a responsible factor. Based on our structural comparison, it is therefore conceivable that dissimilarities in shapes and electrostatic charges of the binding pocket due to highly diverse amino acid compositions within α1, the β-sheet core and the variable loop connecting β2 and β3 might likely be accountable for different nucleotide-binding characteristics. Changes in amino acids in this region responsible for interacting with nucleobase moieties have been proposed previously to strongly influence the cavities of the nucleotide binding cleft and in essence intermolecular bonding, which form a determining factor for affinities and preferences of nucleotide partners^31,36,37^. Such intrinsic properties of KH1-4 might feasibly be fundamental for determining specificity, potentially necessary for the proposed cooperativity and hierarchical mechanisms^6,31^. Nevertheless, the presence of four KH motifs in FUBP1 is likely important for FUSE interaction with high affinity.

## Methods

### Protein expression and purification

The truncated FUBP1 single KH domains, KH1, KH2, KH3 and KH4, the tandem paired proteins, KH1-2, KH2-3 and KH3-4, and the full KH1-4 FUBP1 were subcloned into pNIC28-Bsa4. All recombinant proteins harbouring an N-terminal His_6_ tag were overexpressed in *E. coli* BL21(DE3)-R3-pRARE2*.* Cells cultured in TB media were initially grown at 37 °C to an OD_600_ of 1.6–1.8, and subsequently cooled to 18 °C and at the OD_600_ of ~ 2.6–2.8 induced with 0.5 mM IPTG overnight. Harvested cells were resuspended in a buffer containing 50 mM HEPES, pH 7.5, 500 mM NaCl, 20 mM imidazole, 5% glycerol and 1 mM tris(2-carboxyethyl)phosphine (TCEP), and lysed by sonication. The recombinant proteins were initially purified by Ni^2+^-affinity chromatography. The His_6_ tag was removed by TEV protease treatment, and the cleaved proteins were passed through Ni^2+^ beads and further purified by size exclusion chromatography using Superdex s75 column. The purified proteins were stored in 25 mM HEPES, pH 7.5, 150 mM NaCl, 0.5 mM TCEP. All mutants were generated by PCR-based site-directed mutagenesis, and the mutant protein was expressed and purified using the same procedure.

### Crystallisation, data collection, and structure determination

The recombinant KH2, KH3 and KH4 proteins were concentrated to ~ 10 mg/ml, and crystallized using sitting-drop vapour diffusion method at 20 °C. The crystals of each KH domain were obtained using different reservoir solutions; i) 2 M ammonium sulfate, 5% (v/v) 2-propanol, and 2.5% (v/v) glycerol for KH2, ii) 10% (w/v) PEG 6,000, 10% (v/v) ethylene glycerol, 0.015 M ZnCl_2_ and 0.1 M MES, pH 6.0 for KH3, and iii) 3.1 M sodium formate for KH4. Viable crystals were cryoprotected in mother liquor supplemented with 20% ethylene glycol before flash cooled in liquid nitrogen. Diffraction data were collected at SLS X06SA and BESSY II. Data were processed with XDS^38^ and scaled with aimless^39^. All structures were initially solved by molecular replacement using PHASER^40^ and the structure of KH1 (PDB ID: 4lij). Manual model rebuilding was performed using COOT^41^ and the structures were refined using REFMAC^42^. The final models were verified for geometry correctness with Molprobity^43^. Data collection and refinement statistics are summarized in Supplementary Table S1.

### Native PAGE mobility shift assays

The His_6_-tag-cleaved KH1, KH2, KH3 and His_6_-tagged KH4 recombinant proteins at 200 µM in 25 mM HEPES, pH 7.5, 50 mM NaCl and 0.5 mM TCEP were incubated with a fivefold molar excess of HPSF-purified ssDNA (Eurofins) at 4 °C for 1 h. The mixtures were then mixed with loading dye (0.02% bromophenol blue, 5% glycerol, 0.5× TBE) and loaded onto 10% native polyacrylamide gels. Native PAGE was performed in 0.5× TBE buffer for KH1, KH2 and KH3 or in 0.5× TAE buffer for KH4 at 80 V for 2 h.

### Isothermal titration calorimetry

Isothermal titration calorimetry (ITC) experiments were performed using NanoITC instrument (TA Instrument) at 20 °C in the buffer containing 25 mM HEPES, pH7.5, 50 mM NaCl and 0.5 mM TCEP. The proteins at 0.5–0.8 mM was titrated into the reaction cell containing ssDNA at 0.03–0.05 mM. For tandem paired and full KH domains, the proteins at 0.1–0.25 mM was titrated into the reaction cell containing ssDNA at 0.01–0.02 mM in the buffer of 25 mM HEPES, pH7.5, 150 mM NaCl and 0.5 mM TCEP. The heat of the protein-ssDNA titrations was corrected for the heat of protein dilution, which was calculated from the protein-into-buffer titration experiment. The corrected data were fitted to an independent single binding site model based on the manufacture protocol, from which thermodynamics parameters (ΔH, TΔS and ΔG), equilibrium association and dissociation constants (K_a_ and K_D_) and stoichiometry (n) were calculated.

## Supplementary information

Supplementary information.

## Data Availability

The coordinates and structure factors of all complexes have been deposited to the protein data bank under accession codes 6Y2D, 6Y2C and 6Y24.
