# Supplementary material for: Comparative structural analyses and nucleotide-binding characterization of the four KH domains of FUBP1
Source: Sci Rep. 2020 Aug 10;10:13459. doi: 10.1038/s41598-020-69832-z (PMC7417555; doi:10.1038/s41598-020-69832-z)
Supplement: Supplementary file 1 — Supplementary information. [file 41598_2020_69832_MOESM1_ESM.pdf]

Comparative structural analyses and nucleotide-binding characterization of the four KH domains of FUBP1

Xiaomin Ni[1,2], *Stefan Knapp*[1,2,3*], *Apirat Chaikuad*[1,2*]

[1] Institute of Pharmaceutical Chemistry, Goethe-University Frankfurt, 60438 Frankfurt, Germany

[2] Structural Genomics Consortium, BMLS, Goethe-University Frankfurt, 60438 Frankfurt, Germany

[3] German translational cancer network (DKTK), Frankfurt/Mainz site, 60438 Frankfurt, Germany

Supporting Information

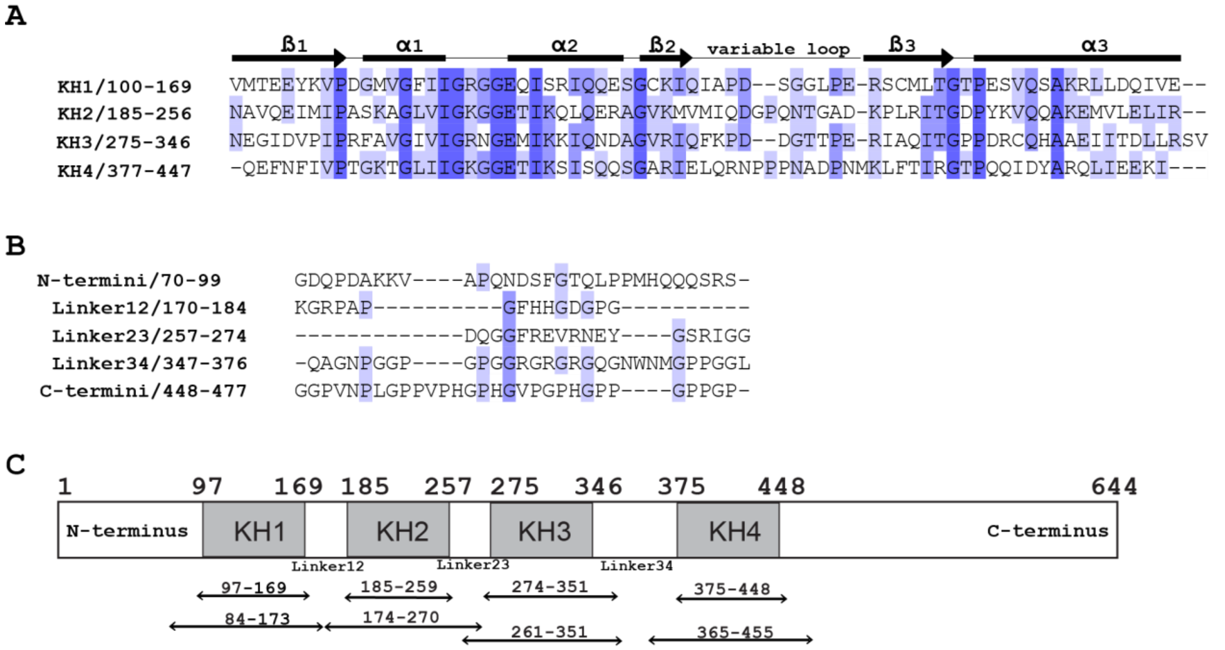

Supplementary Figure S1. Sequence comparison of FUBP1 KH1-4. Sequence alignments of the central core of KH1-4 with predicted secondary structure (A) and the linker region between domains (B). Conserved amino acids are highlighted in blue. C) Schematic illustration of truncated constructs for the four KH domains. The constructs leading used for crystallization and ssDNA-binding characterization are shown in Figure 1.

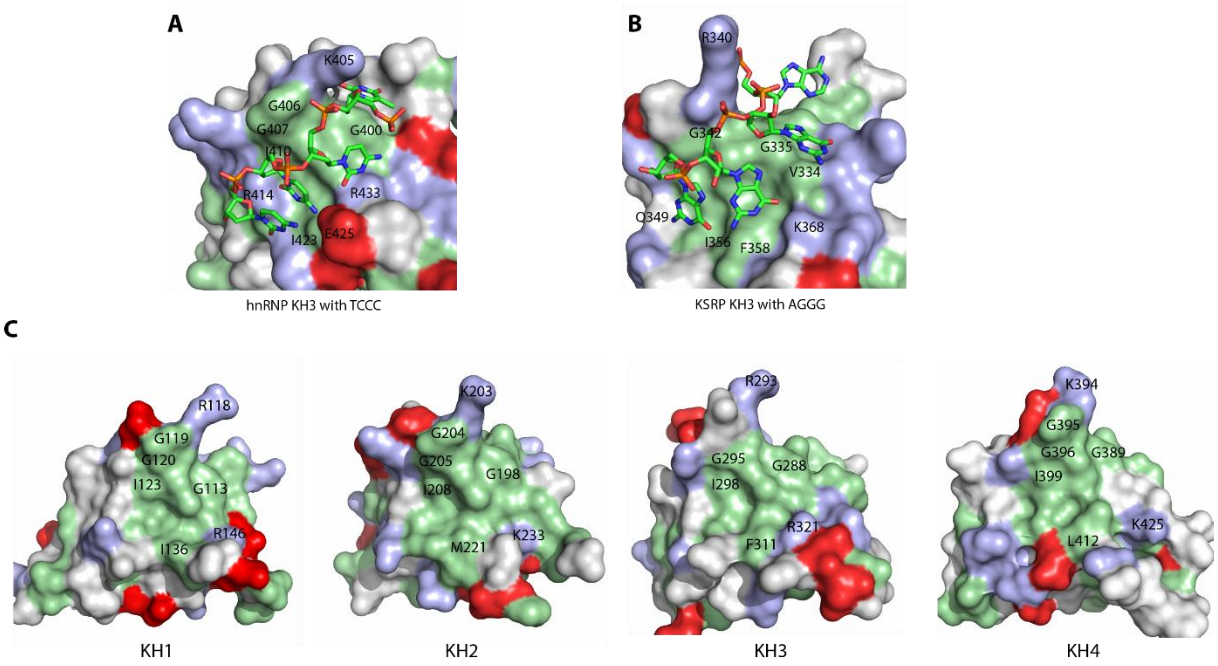

Supplementary Figure S2. Mapping of the GXXG motif and hydrophobic residues that define the upper part of the nucleotide binding cleft responsible for interaction with nucleotide backbone. Shown are surface representation of the binding pocket of hnRNP K KH3-ssDNA (PDB code: 1zzi) (A), KSRP (FUBP2) KH3-ssDNA (PDB code: 4b8t) (B) and FUBP1 KH1-4 (C). Positively-charged, negatively-charged and hydrophobic residues are colored in blue, red and green, respectively. Figures were created using PyMOL software (https://pymol.org/)

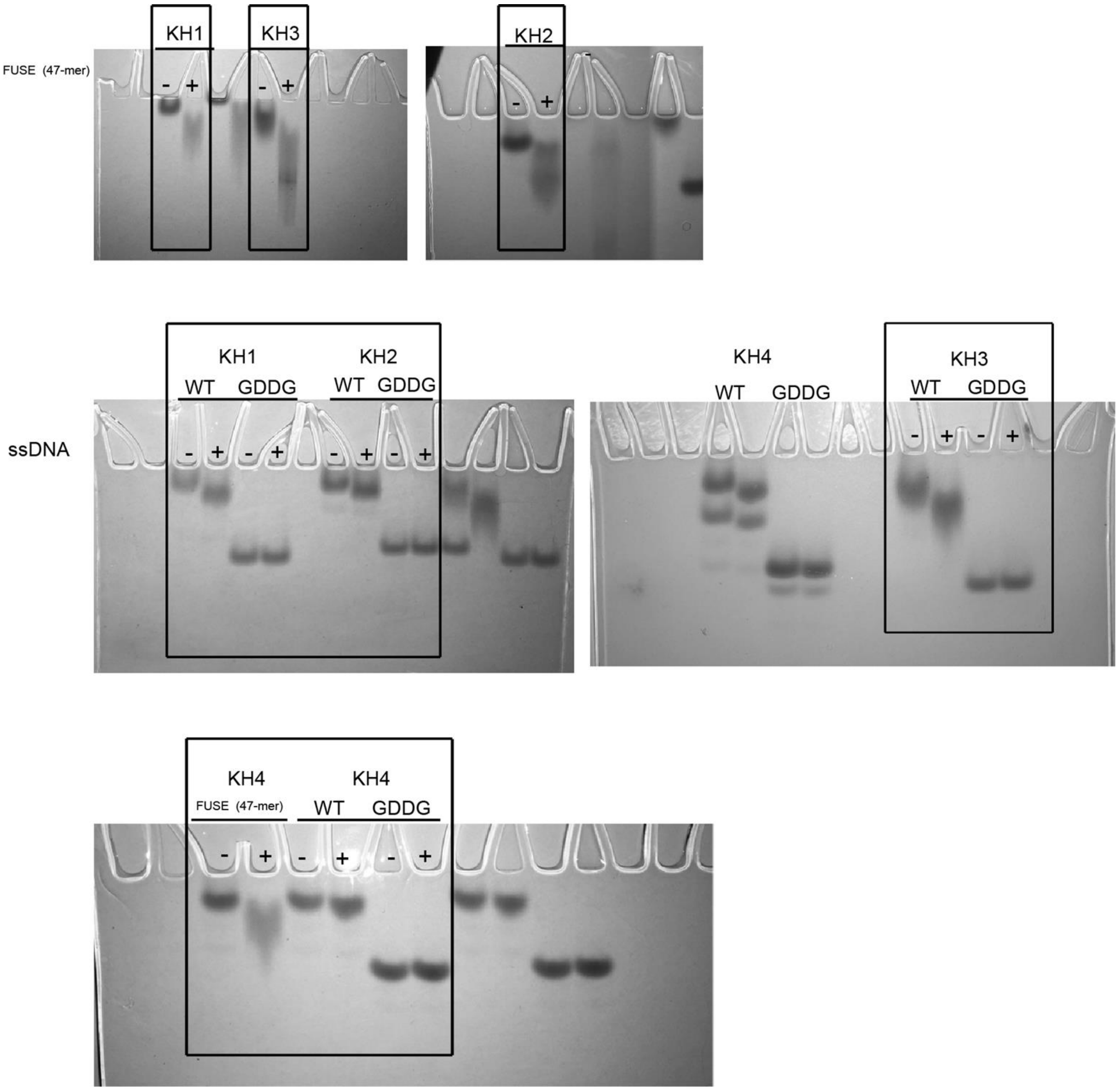

Supplementary Figure S3a: NativePAGE gels (marked in box) used for generating Figure2A and C. KH4 appeared as double bands (top right gel) when running in TBE (Tris-Boric Acid-EDTA) but not in TAE (Tris-Acetic Acid-EDTA) as shown in bottom gel.

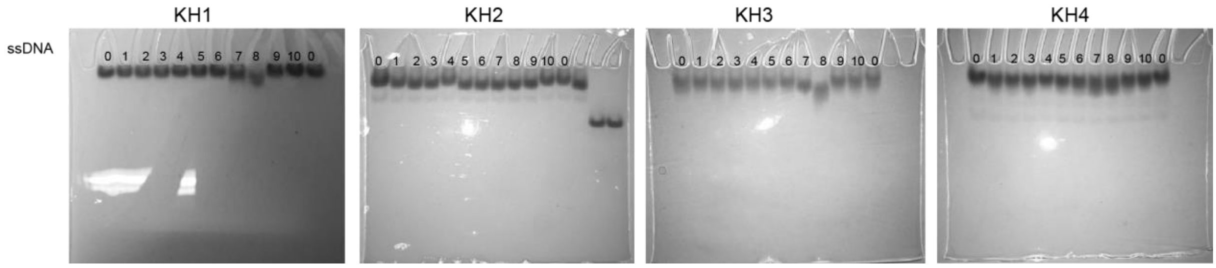

Supplementary Figure S3b: NativePAGE gels (running in TBE) used to generate Figure2B.

Supplementary table S1. Data collection and refinement statistics

Protein	KH2	KH3	KH4
PDB accession code	6Y2D	6Y2C	6Y24
Data Collection			
Resolution[a] (Å)	49.37-1.90 (1.94-1.90)	49.30-2.00 (2.05-2.00)	55.04-1.86 (1.90-1.86)
Spacegroup	$P\ 2_1$	$P\ 3_221$	$C\ 222_1$
Cell dimensions	a =28.9, b = 82.2, c = 49.4 Å	a = b = 47.4, c = 147.9 Å	a =70.0, b = 89.1, c = 28.7 Å
	$\alpha = \gamma$ = 90.0° , β = 91.0°	$\alpha = \beta$ = 90.0°, γ = 120.0°	α, β, γ = 90.0°
No. unique reflections[a]	18,030 (1,152)	13,595 (999)	7,912 (486)
Completeness[a] (%)	99.0 (99.1)	98.8 (99.2)	100.0 (100.0)
I/σI[a]	5.3 (1.7)	8.3 (2.4)	9.4 (1.8)
R_{merge}[a] (%)	13.2 (82.7)	9.2 (51.6)	9.5 (81.1)
CC (1/2)	0.990 (0.719)	0.995 (0.661)	0.996 (0.779)
Redundancy[a]	3.9 (4.0)	4.5 (4.6)	6.1 (5.6)
Refinement			
No. atoms in refinement (P/L/O)[b]	2,089/ 0/ 126	1,193/ 0/ 63	614/ 0/ 38
B factor (P/L/O)[b] ($Å^2$)	25/ 0/ 38	38/ 0/ 40	43/ 0/ 44
R_{fact} (%)	19.5	20.3	19.3
R_{free} (%)	23.9	24.0	24.3
rms deviation bond[c] (Å)	0.016	0.015	0.016
rms deviation angle[c] (°)	1.7	1.5	1.5
Molprobity Ramachandran			
Favour (%)	98.16	98.68	100.00
Outlier (%)	0	0	0

[a] Values in brackets show the statistics for the highest resolution shells.

[b] P/L/O indicate protein, ligand molecules, and other (water and solvent molecules), respectively.

[c] rms indicates root-mean-square.
